# The intravascular volume effect of Ringer's lactate is below 20%: a prospective study in humans

**DOI:** 10.1186/cc11344

**Published:** 2012-05-16

**Authors:** Matthias Jacob, Daniel Chappell, Klaus Hofmann-Kiefer, Tobias Helfen, Anna Schuelke, Barbara Jacob, Alexander Burges, Peter Conzen, Markus Rehm

**Affiliations:** 1 Department of Anaesthesiology, University Hospital Munich, Nussbaumstrasse 20, 80336 Munich, Germany; 2 Department of Gynecology and Obstetrics, University Hospital Munich, Marchioninistrasse 15, 81377 Munich, Germany

## Abstract

**Introduction:**

Isotonic crystalloids play a central role in perioperative fluid management. Isooncotic preparations of colloids (for example, human albumin or hydroxyethyl starch) remain nearly completely intravascular when infused to compensate for acute blood losses. Recent data were interpreted to indicate a comparable intravascular volume effect for crystalloids, challenging the occasionally suggested advantage of using colloids to treat hypovolemia. General physiological knowledge and clinical experience, however, suggest otherwise.

**Methods:**

In a prospective study, double-tracer blood volume measurements were performed before and after intended normovolemic hemodilution in ten female adults, simultaneously substituting the three-fold amount of withdrawn blood with Ringer's lactate. Any originated deficits were substituted with half the volume of 20% human albumin, followed by a further assessment of blood volume. To assess significance between the measurements, repeated measures analysis of variance (ANOVA) according to Fisher were performed. If significant results were shown, paired *t *tests (according to Student) for the singular measurements were taken. *P *< 0.05 was considered to be significant.

**Results:**

A total of 1,097 ± 285 ml of whole blood were withdrawn (641 ± 155 ml/m^2 ^body surface area) and simultaneously replaced by 3,430 ± 806 ml of Ringer's lactate. All patients showed a significant decrease in blood volume after hemodilution (-459 ± 185 ml; *P *< 0.05) that did not involve relevant hemodynamical changes, and a significant increase in interstitial water content (+2,157 ± 606 ml; *P *< 0.05). The volume effect of Ringer's lactate was 17 ± 10%. The infusion of 245 ± 64 ml of 20% human albumin in this situation restored blood volume back to baseline values, the volume effect being 184 ± 63%.

**Conclusions:**

Substitution of isolated intravascular deficits in cardiopulmonary healthy adults with the three-fold amount of Ringer's lactate impedes maintenance of intravascular normovolemia. The main side effect was an impressive interstitial fluid accumulation, which was partly restored by the intravenous infusion of 20% human albumin. We recommend to substitute the five-fold amount of crystalloids or to use an isooncotic preparation in the face of acute bleeding in patients where edema prevention might be advantageous.

## Introduction

Fluid homeostasis within the human body is the result of complex interactions between compartments and barriers and the prerequisite of stable hemodynamics [1]. Severe intravascular hypovolemia is undesirable, being associated with a decreased cardiac output and leading to tissue hypoperfusion and organ dysfunction [2,3]. Therefore, perioperative infusion therapy should avoid these complications by replacing blood and fluid losses as timely and adequately as possible. However, discussions on the adequate type, composition and amount of fluids are ongoing [2,4,5]. While replacement of crystalloidal extracellular losses, such as urinary output and insensible perspiration, using isotonic crystalloids seems rational [2], there are controversial opinions for maintaining or restoring cardiac preload [6]. Theoretical physiological considerations on vascular barrier properties may suggest a possible advantage of artificial colloids or natural proteins: iso-oncotic preparations are believed to remain within the intravascular space while crystalloids should distribute evenly across the whole extracellular compartment, that is, to 80% interstitially [2]. Surprisingly, a prospective randomized trial on 6,997 critically ill patients showed no differences in mortality or organ dysfunction when using either human albumin or crystalloids for resuscitation guided by common hemodynamical surrogates [7]. Furthermore, the authors observed only a slight difference of the total infused amount of the respective solutions. Despite the fact that these patients might have had, at least partly, an altered vascular barrier, one important question increasingly arose: is our historical belief in a superior volume effect of colloids in comparison to crystalloids just a physiological myth that should urgently be adapted to clinical reality?

This study assessed, in the highly standardized setup of preoperative acute normovolemic hemodilution [8] combined with repeated direct blood volume assessments, the actual volume effect of an isotonic crystalloid when applied as a substitute of artificial acute blood loss in patients. Presumably, the vascular barrier primarily was sufficient and endothelial and capillary functioning was normal in our model. We hypothesized a relevant (> 10%) and significant decrease in blood volume when using Ringer's lactate for this indication, the primary study endpoint being blood volume (ml) after hemodilution. Beyond that, the capacity of hyperoncotic (20%) human albumin to recruit interstitially lost fluid for cardiac preload was investigated.

## Materials and methods

The study was approved by the ethics committee of our institution (Ethikkommission der Medizinischen Fakultät der Ludwig-Maximilians-Universität München, File number 022-06). All patients gave written informed consent. A prospectively performed power analysis (for details see Appendix 1) required a total inclusion of ten patients. All patients had no cardiovascular or pulmonary dysfunctions (American Society of Anesthesiologists physical status I to II) and were scheduled for radical hysterectomy because of a carcinoma of the cervix. All measurements were performed under general anesthesia to ensure steady-state conditions and to avoid the unpleasant procedure of inserting central venous and arterial catheters into an awake patient.

### Anesthesiological procedure

After arrival in the operating theatre, monitoring and a peripheral canula were applied and 60 ml of whole blood were withdrawn for preparation of two red cell volume (RCV) measurements (see below). A thoracic epidural catheter was inserted; however, local anesthetics were only infused after having completed the study protocol. General anesthesia was induced with sufentanil, propofol, and cisatracurium, and, after tracheal intubation, maintained via a continuous propofol infusion and repeated sufentanil injections. Mechanical ventilation with inspiratory oxygen content of 0.4 was guided to maintain arterial carbon dioxide partial pressure at approximately 40 mmHg. A second peripheral canula, a femoral arterial, a central venous and a urine catheter were inserted. Until the end of the study procedure (see below), no additional intravenous infusions were applied, except for negligible amounts that were necessary to inject the intravenous drugs. Continuous monitoring included electrocardiogram, direct arterial blood pressure, pulse oximetry, determinations of hematocrit and hemoglobin concentration, and arterial blood gas analyses.

### Study protocol

**Figure 1:**
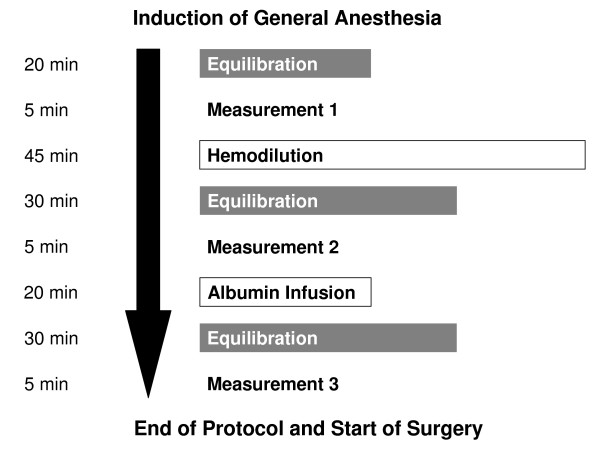
**Time frame of study procedures including the three measurements between induction of general anesthesia and start of surgery**.

Following induction of general anesthesia, stable hemodynamics were initiated during a time interval of at least 20 minutes after which the baseline blood volume (BV) measurement (measurement 1) was performed. After hemodilution (see Hemodilution procedure) a 30 minutes break re-ensured hemodynamical steady-state conditions before the next BV measurement was carried out (measurement 2). In case blood volume decreased in comparison to baseline measurement 1, half of the deficit was replaced with 20% human albumin (CSL Behring GmbH, Marburg, Germany). Plasma volume (PV) was then reassessed (measurement 3) following a repeated 30-minute break for equilibration. RCV has been shown to remain unchanged if no transfusion or withdrawal of red cells occurred [9]. Simultaneous to each measurement, hematocrit and cumulative urine production were recorded (Figure 1). Norepinephrine was applied intravenously throughout the study phase to maintain the mean arterial blood pressure above 60 mmHg.

### Hemodilution procedure

Hemodilution was intended to be normovolemic, that is, to maintain the intravascular volume state. Blood was removed at a rate of about 25 to 30 ml/min via the arterial and the central venous lines and simultaneously replaced with Ringer's lactate (B. Braun Melsungen AG, Melsungen, Germany) at the three-fold amount via peripheral canulas. According to previous studies on volume effects performing acute normovolemic hemodilution (ANH) as a model situation, it was intended to withdraw about 700 ml/m^2 ^body surface area (BSA) of whole blood to simulate significant bleeding without falling below a hematocrit of 0.24 [9]. The hemodilution bags were weighed on a precision scale so that the withdrawn blood volume could be evaluated online. Also, hematocrit was frequently measured to respect the lower threshold. The infused amount of Ringer's lactate (three-fold the loss) was a practicable compromise between the physiological expectations suggesting the five-fold amount and recent suggestions from literature (1.4-fold) [7].

### Direct Measurements

#### Nonradioactive assessment of blood volume components

PV was determined by diluting indocyanine green (ICG) using the whole blood method [10-12]. In brief, after calibration of the PV measurement system, we injected a bolus of the dye via the central venous line. The target was calculated by extrapolating the theoretical dilution of ICG at injection time from several timed measurements. Simultaneously, we measured RCV using sodium fluorescein-labeled autologous red cells, injecting them via the central venous line. From the dilution, determined by flow cytometry, we calculated total body RCV, according to Lauermann and coworkers [13].

For methodological details see Appendix 2.

#### Determination of hematocrit

Hematocrit was measured in triplicate without correction for plasma trapping by centrifugation. In discrimination to the calculated whole body hematocrit (HK_WB_, see below) this value, directly determined in samples gained from large vessels, will be termed the large vessel hematocrit (HK_LV_) in the following.

### Further major calculations

An exact value of blood volume results from adding the directly measured red cell and plasma volumes:

(E1) $$\text{BV}\left(\text{ml}\right)=\text{RCV}\left(\text{ml}\right)+\text{PV}\left(\text{ml}\right)$$

The blood content of ANH bags (BC_bag_) was calculated as:

(E2) $$\begin{array}{l} {\text{BC}}_{\text{bag}}(\text{ml})\\ \;=\left(\text{weight of full ANH bags}-110\text{ g}\right)/1.05\text{ g}/\text{ml} \end{array}$$

with 110 g being the sum of the *c*itrate phosphate dextrose adenine (CPDA-1) fluid content (70 g) and the weight of empty ANH bags (40 g). The specific gravity of blood was assumed to be 1.05 g/ml [14].

The red cell content of ANH bags (RCC_bag_) was calculated according to:

(E3) $$\text{RC}{\text{C}}_{\text{bag}}\left(\text{ml}\right)=\left(\text{B}{\text{C}}_{\text{bag}}\left(\text{ml}\right)+70\text{ ml}\right)\times \text{H}{\text{K}}_{\text{bag}}$$

The theoretical whole body hematocrit (HK_WB_) represents a calculated mean hematocrit throughout the circulation, calculated from quantified blood components:

(E4) $$\text{H}{\text{K}}_{\text{WB}}=\text{RCV}\left(\text{ml}\right)\times \text{BV}{\left(\text{ml}\right)}^{-1}$$

HK_WB _is normally slightly lower than large vessel hematocrit (HK_LV_) measured in blood samples in clinical routine. This phenomenon is reflected by an F_cell _ratio, normally being smaller than one [15,16]:

(E5) $${\text{F}}_{\text{cell}}=\text{H}{\text{K}}_{\text{WB}}\times \text{H}{\text{K}}_{\text{L}{\text{V}}^{-1}}$$

The reason is most likely the existence of the endothelial glycocalyx, which binds plasma proteins to the endothelial surface [17]. This functionally divides the directly measured total PV into circulating plasma volume (PV_circ_) and a noncirculating part representing the total volume of the endothelial surface layer (ESL).

(E6) $$\text{P}{\text{V}}_{\text{circ}}\left(\text{ml}\right)=\left(\text{RCV}\left(\text{ml}\right)\times \left(1-\text{H}{\text{K}}_{\text{LV}}\right)\right)\times \text{H}{\text{K}}_{\text{L}{\text{V}}^{-1}}$$

(E7) $$\text{ESL}\left(\text{ml}\right)=\text{PV}\left(\text{ml}\right)-\text{P}{\text{V}}_{\text{circ}}\left(\text{ml}\right)$$

The presumable cumulative increase in interstitial water content after ANH (IE_ANH_) and after protein infusion (IE_PI_) vs. baseline was calculated as follows:

(E8) $$\text{I}{\text{E}}_{\text{ANH}}\left(\text{ml}\right)=\left(\text{CI}\left(\text{ml}\right)-\text{BW}\left(\text{ml}\right)-\text{U}{\text{O}}_{\text{ANH}}\left(\text{ml}\right)\right)+\left(\text{B}{\text{V}}_{1}\left(\text{ml}\right)-\text{B}{\text{V}}_{2}\left(\text{ml}\right)\right)$$

CI, crystalloidal infusion; BW, blood withdrawal; UO_ANH_, urinary output during ANH procedure; BV_1_, blood volume before ANH; BV_2_, blood volume after ANH

(E9) $$\text{I}{\text{E}}_{\text{PI}}\left(\text{ml}\right)=\text{I}{\text{E}}_{\text{ANH}}\left(\text{ml}\right)-\left(\text{B}{\text{V}}_{3}\left(\text{ml}\right)-\text{B}{\text{V}}_{2}\left(\text{ml}\right)\right)+\text{PI}\left(\text{ml}\right)-\text{U}{\text{O}}_{\text{PI}}\left(\text{ml}\right)$$

BV_3_, blood volume after protein infusion; PI, protein infusion; UO_PI_, urinary output during protein infusion

The initial volume effect of Ringer's lactate (VE_rl_) and 20% human albumin (VE_ha_), that is, that part remaining within the vasculature for at least 30 minutes after the end of hemodilution, including a potential additional volume recruited from the interstitial space, was calculated according to:

(E10) $$\text{V}{\text{E}}_{\text{rl}}\left(\%\right)=\left(\left(\left(\text{B}{\text{V}}_{2}\left(\text{ml}\right)-\text{B}{\text{V}}_{1}\left(\text{ml}\right)\right)+\text{BW}\left(\text{ml}\right)\right)\times \text{CI}{\left(\text{ml}\right)}^{-1}\right)\times 100$$

BV_2_, blood volume after ANH; BV_1_, blood volume before ANH; BW, blood withdrawal; CI, crystalloidal infusion

(E11) $$\text{V}{\text{E}}_{\text{ha}}\left(\%\right)=\left(\left(\text{B}{\text{V}}_{3}\left(\text{ml}\right)-\text{B}{\text{V}}_{2}\left(\text{ml}\right)\right)\times \text{PI }{\left(\text{ml}\right)}^{-1}\right)\times 100$$

BV_3_, blood volume after protein infusion; PI, protein infusion

### Statistics

A detailed statistical analysis is attached in Appendix 1. The power analyses were performed using the free G*Power software, version 3.1.

All data are presented as mean ± standard deviation (SD), with n indicating the number of patients. All variables were tested for normality with the Kolmogorov-Smirnov test and homoscedasticity (homogeneity of variances) with Levene's test. For comparison of multiple measurements, repeated measures analyses of variance (ANOVA) according to Fisher were performed. If significant results were shown, paired *t *tests (according to Student) for the singular measurements were taken. *P *< 0.05 was considered to be significant. The statistical software used to conduct the analyses was SPSS 18 (SPSS Inc., Chicago, IL, USA).

## Results

The demographic characteristics of the patients can be taken from Table 1 (for calculations see Appendix 3). Measured and calculated variables before (measurement 1) and after (measurement 2) the hemodilution procedure, as well as after albumin infusion (measurement 3) are shown in Table 2. During hemodilution 1,097 ± 285 ml of whole blood (641 ± 155 ml/m^2^), including 347 ± 99 ml of red cells, were withdrawn and replaced simultaneously by 3,430 ± 806 ml of Ringer's lactate. This led to a significant decrease of the total blood volume by 459 ± 185 ml vs. baseline, the volume effect of the crystalloidal preparation being 17 ± 10%. Replacement of this deficit with 245 ± 64 ml of 20% human albumin led to an almost complete restoration of blood volume, the slight remaining deficit in comparison to measurement 1 was 25 ± 271 ml. Accordingly, the calculated volume effect was 184 ± 63% for the hyperoncotic protein solution. While replacing the blood loss with crystalloids caused a significant increase of tissue water content, remobilization of this interstitial edema by hyperoncotic protein infusion was partly possible, but inter-individually quite inconstant (438 ± 299 ml). The hemodilution procedure led to a significant decrease in the total volume of the endothelial surface layer by 240 ± 133 ml, which could not be restored by protein infusion.

**Table 1:** Patient characteristics (*n *= 10).

Age (yr)	46 ± 11
Height (cm)	165 ± 4
Weight (kg)	65 ± 14
BSA (m^2^)	1.71 ± 0.17
BMI (kg × m^-2^)	23.7 ± 5.1

Values are mean ± standard deviation (SD). BMI, body mass index; BSA, body surface area (for calculations see Appendix 3).

**Table 2:** Measured and calculated variables (*n *= 10).

	Measurement 1	Measurement 2	Measurement 3
	(Before hemodilution)	(After hemodilution)	(After protein infusion)
RCV (ml)	1,273 ± 249¶	947 ± 195§	947 ± 195*
PV (ml)	2,686 ± 297║	2,554 ± 378║	2,987 ± 408‡
BV (ml)	3,959 ± 387¶	3,501 ± 499‡	3,934 ± 500¶
HK_WB_	0.32 ± 0.05‡	0.27 ± 0.04‡	0.24 ± 0.04‡
HK_LV_	0.35 ± 0.04‡	0.28 ± 0.04‡	0.25 ± 0.04‡
F_cell_	0.91 ± 0.03‡	0.97 ± 0.02§	0.97 ± 0.02§
ESL (ml)	339 ± 130‡	99 ± 54§	120 ± 77§
Cumulative urine production# (ml)		635 ± 200†	884 ± 171†
Increase in interstitial water content# (ml)		2,157 ± 606†	1,719 ± 680†

*Not reassessed, but taken from measurement 2 (no further blood withdrawal), #since the beginning of hemodilution procedure (for calculations see Materials and Methods section), §*P *< 0.05 vs. measurement 1, ¶*P *< 0.05 vs. measurement 2, ║*P *< 0.05 vs. measurement 3, ‡*P *< 0.05 vs. both other measurements, †*P *< 0.05 vs. basal and other measurement, respectively. For further details on the statistical analysis see Appendix 1. Values are mean ± standard deviation (SD). BV, blood volume; ESL, volume of the endothelial surface layer; ${\text{F}}_{\text{cell}}\text{,}\;\text{H}{\text{K}}_{\text{WB}}\times {\text{HK}}_{{\text{LV}}^{-1}}$; HK_LV_, large vessel hematocrit; HK_WB_, whole body hematocrit; PV, plasma volume; RCV, red cell volume.

**Figure 2:**
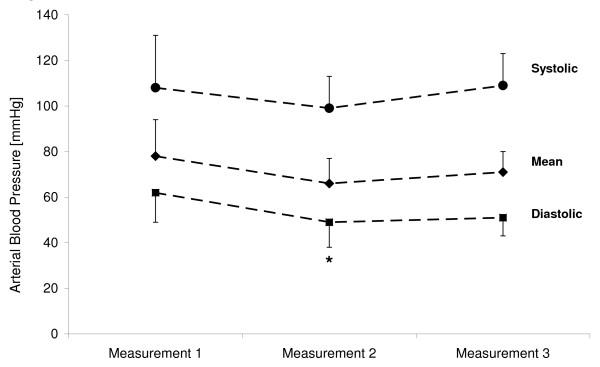
**Systolic, mean and diastolic arterial blood pressure at measurements 1, 2 and 3, respectively**. *P < 0.05 vs. measurement 1.

The systolic, mean and diastolic arterial blood pressure are graphically outlined in Figure 2. No statistically significant changes of systolic and mean arterial blood pressures nor heart rate were observed throughout the study procedure, the heart rate having been 62 ± 14, 58 ± 7 and 55 ± 8 beats per minute (bpm) at measurements 1, 2 and 3, respectively (*P *> 0.05). Norepinephrine dosage after induction of general anesthesia (measurement 1) was 0.1 ± 0.1 mg/hour. There were no significant adjustments until the end of the study procedure. No patient received more than 0.2 mg/hour norepinephrine during any phase of the study. There was no difference of diastolic blood pressures between measurements 2 and 3 (*P *> 0.05), while a significant difference between measurements 1 and 2 was observed (*P *< 0.05).

## Discussion

In this prospective investigation on patients being primarily in a cardiocirculatory steady-state, all participants were significantly hypovolemic compared to the starting point after an acute blood loss and simultaneous substitution with the three-fold amount of Ringer's lactate. This effect was associated with a significant fluid storage outside the circulation and a marked reduction of an important structure concerning vascular barrier functioning. The shift outward by more than 80% of the infused amount of crystalloid was completed within 30 minutes after the end of the hemodilution, being much faster than generally assumed.

Normovolemic hemodilution is a well established blood conservation technique, reducing the requirement for allogeneic red cell transfusions [8]. Autologous blood is safely stored outside the circulation prior to elective surgery and retransfused if required. During withdrawal, the whole blood is replaced simultaneously by an artificial solution in order to maintain the quantitative component of cardiac preload [18]. From a scientific point of view, this preoperative intervention can be comprised as simulation of accidental bleeding and therapeutical infusion therapy. The intravascular volume effect of the chosen substitute, that is, that part remaining within the circulation after intravenous infusion, determines the required amount to establish or maintain intravascular normovolemia [19]. A volume effect of, for example, 100% means that substitution of an equal amount as withdrawn is necessary, whereas an effect of 50% would require double the amount. In the latter case, the excessive part of the infused amount is excreted via the kidneys and/or shifted toward the interstitial space [19].

**Figure 3:**
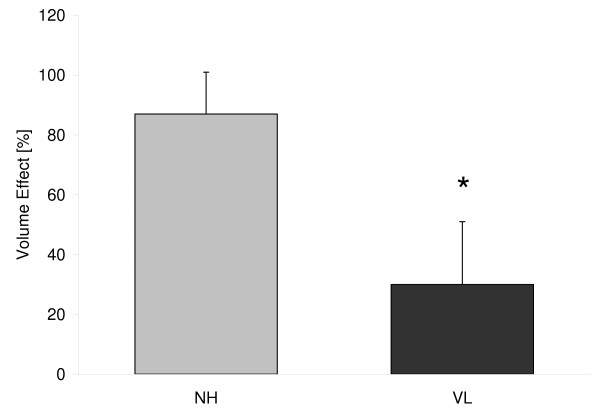
**The different volume effects of an isooncotic albumin preparation during volume loading (VL, *n *= 10) **[12]**and normovolemic hemodilution (NH, *n *= 15) **[9]**assessed by double-tracer measurements in healthy female patients**. *P < 0.05.

Numerous volume effects of colloids using various assessment techniques have been published before, being, except for 4% gelatine, constantly around 100% (Table 3) [11,20-26]. As to be expected, only hypertonic preparations showed an increase of the intravascular compartment by significantly more than the infused amount [24,25]. Obviously, they were able to recruit fluids from the extravascular towards the circulatory space. Beyond that, especially direct double-tracer measurements that compared overloading a normovolemic circulation (hypervolemia) to replacement of acute blood losses have revealed that colloidal volume effects might not be consistent, but context-sensitive (Figure 3) [19]. To our knowledge, only two studies have considered the volume effect of a hypervolemic crystalloidal bolus, without simultaneous blood withdrawal. Estimations around 20% were based on indirect calculations involving the large vessel hematocrit measured 60 minutes after bolus infusion [23] or after a slow infusion of one liter over 90 minutes [27]. The intravascular persistence of isotonic crystalloid boluses to substitute an acute blood loss has repeatedly been assumed to be much higher [28], but has not been addressed before.

**Table 3:** Volume effects of colloids in literature.

Preparation	[%]	n	Reference
4% Human Albumin	83	9	[20]
5% Human Albumin	99	6	[26]
20% Human Albumin	250	26	[24]

6% HES 130/0.4	98	10	[11]
6% HES 200/0.62	93	9	[20]
6% HES 450/0.7	120	10	[21]
10% HES 200/0.5	109	10	[22]
14% HES 200/0.5	247	6	[25]

6% Dextran 70	79	14	[27]

4% Gelatine	58	10	[23]

Values are mean volume effects. HES, hydroxyethyl starch; n, number of patients.

Directly assessing the entire blood volume using a double-tracer approach, as performed here, is time-, personal- and equipment-consuming and, therefore, currently not available for clinical routine. However, recent physiological insights suggest that it might be the only reliable approach to answer scientific questions around volume effects, mainly for methodological reasons. A healthy vascular endothelium is covered by a microscopical structure consisting of the endothelial glycocalyx and bound plasma constituents, mainly proteins [17,29]. This so-called endothelial surface layer, a presumably important structure concerning vascular integrity [29], represents an exclusion zone for red cells. Accordingly, the peripherally measured large vessel hematocrit does not reflect the mean composition of the total blood volume, but only that of the freely circulating part [10]. The observed reduction of the total volume of the endothelial surface layer during ANH caused an additional decrease in large vessel hematocrit (HK_LV_) beyond that reflecting a net loss of red cells, simply by increasing their distribution space. This phenomenon was most likely not an effect of the applied substance itself, but of intravascular hypervolemia [12]. Obviously, even the short-term effect preceding the distribution of Ringer's lactate over the entire extracellular compartment appears enough to cause these significant alterations at the endothelial surface, leading to an overestimation of the volume effect when based on blood volume calculations using hematocrit.

Showing an intravascular volume effect for Ringer's lactate of 17 ± 10% while replacing an acute blood loss, the present data coincide not only with physiological considerations, but also with clinical observations. It is interesting to physiologists, but startling to clinicians that the significant intravascular hypovolemia resulting from our abortive attempt of normovolemic hemodilution with crystalloid was not reflected by any clinically relevant hemodynamical changes or vasopressor requirements. This, however, confirms very recent functional observations in patients before elective surgery [30] and might be an explanation of what was observed in a highly cited large prospective clinical trial on a mixed collective of 6,997 ICU patients [7]. The study compared resuscitation with isotonic saline vs. an isooncotic human albumin preparation under routine monitoring conditions. The patients of the crystalloid group received in total only the 1.4-fold amount of those stabilized with protein solutions. The interpretation to have evidence of a similar volume effect between these two different classes of preparations under intensive care conditions [7] might be, on the one hand, quite justified, as critical illness might significantly contribute to a breakdown of the vascular barrier [29]. On the other hand, however, the clinical impression of cardiocirculatory stability might be put into an interesting new perspective by the present data, despite being derived from a completely different collective under highly standardized conditions: Obviously, at least cardiopulmonary-compensated patients receiving crystalloids to stabilize cardiac preload in the face of acute bleeding are endangered to sustain undetected intravascular hypovolemia under routine monitoring. Such an occult deficit might be of relevance to a patient. It has been reported that compensatory vasoconstriction might negatively influence local tissue perfusion [31]. Already a blood loss of 10% has been demonstrated to relevantly compromise splanchnic perfusion [32]. In our study, the mean decrease in blood volume by 11.6% was most likely accompanied by an impressive interstitial edema of more than two liters. This is not only a short-term effect, but can be relatively lasting and, in addition, negatively influences microcirculation and tissue oxygen tension [2]. Even in healthy volunteers, the kidneys need more than two days to excrete this fluid overload [33]. Postoperative weight gain after fluid overload has been shown to be associated with an increased perioperative mortality [34]. Our applied study protocol most likely created an undesirable patient condition prior to major abdominal surgery.

By trying to recruit the most likely interstitially accumulated water with a 20% human albumin preparation we functionally demonstrated a severely impaired barrier competence after hemodilution: The volume effect of this hyperoncotic preparation was less than half of the theoretical expectation. Nevertheless, being well over 100%, hyperoncotic colloids are an option to recruit interstitially stored fluid toward the circulatory compartment. It is crucial to bear potential side effects of hyperoncotic colloids in mind. Whereas a recent large meta-analysis showed no severe complications for hyperoncotic preparations of the natural human protein albumin [35], artificial hyperoncotic solutions are known to have a negative impact on kidney function [4]. Further prospective clinical trials on this topic are urgently needed to clarify the definite role of this class of preparations.

## Conclusion

To maintain or restore blood volume in the face of acute bleeding, isotonic crystalloids are required in the five-to-six-fold amount of the estimated deficit to reach normovolemia, even if the vascular barrier is intact. The pathophysiological impact of such a volume overload on the organism might be severe. The actual impact on outcome after elective surgery, however, or the transferability of these insights to the critically ill patient remain unclear and must be urgently investigated in future studies.

## Key messages

• Intravascular volume effect of Ringer's lactate is below 20%

• To substitute blood losses with the three-fold amount of Ringer's lactate is insufficient and leads to intravascular hypovolemia

• Intravascular hypovolemia of more than 10% remained undetected by standard hemodynamical monitoring (ECG, online arterial blood pressure)

• 20% human albumin might be able to recruit interstitially stored fluid back towards the circulatory space

## Abbreviations

ANH: acute normovolemic hemodilution; BC_bag_: blood content of ANH bags; BMI: body mass index; BPM: beats per minute; BSA: body surface area; BV: blood volume; BW: blood withdrawal; CI: crystalloidal Infusion; CPDA-1: *c*itrate phosphate dextrose adenine; E: equation; ECG: electrocardiography; ESL: endothelial surface layer; HK_LV_: large vessel hematocrit; HK_WB_: whole body hematocrit; ICG: indocyanine green IE_ANH_: increase in interstitial water content after ANH; IE_PI_: increase in interstitial water content after protein infusion; PI: protein Infusion; PV: plasma volume; PV_circ_: circulating plasma volume; RCC_bag_: red cell content of ANH bags; RCV: red cell volume; UO_ANH_: urinary Output during ANH procedure; UO_PI_: urinary Output during protein Infusion; VE_ha_: volume effect of human albumin; VE_rl_: volume effect of Ringer's lactate

## Competing interests

This study was performed using departmental research funding provided by the Bavarian government (Bayerisches Staatsministerium für Wissenschaft, Forschung und Kunst, München; Bavarian State Ministry of Science, Research and the Arts, Munich). In addition, an unrestricted grant was given to the Department of Anesthesiology, University Hospital Munich by CSL Behring GmbH (Marburg, Germany). It was not linked to any influence on study design or manuscript approval by the CSL Behring company.

MJ states to have held lectures for Baxter Deutschland GmbH (Unterschleißheim, Germany), Fresenius Kabi Deutschland GmbH (Bad Homburg, Germany), B Braun, Melsungen AG (Melsungen, Germany) and Serumwerk Bernburg AG (Bernburg, Germany).

DC has held lectures for Fresenius Kabi Deutschland GmbH (Bad Homburg, Germany).

MR has held lectures for CSL Behring GmbH (Marburg, Germany) and Fresenius Kabi Deutschland GmbH (Bad Homburg, Germany).

For other studies MJ and MR have received grants from Serumwerk Bernburg AG (Bernburg, Germany), CSL Behring GmbH (Marburg, Germany) and Fresenius Kabi Deutschland GmbH (Bad Homburg, Germany).

## Authors' contributions

MJ, DC, PC and MR conceived and designed the study. MJ, DC and MR performed the measurements and acquired the data while KHK performed anesthesia. TH and AS performed the erythrocyte preparation and FACS analysis, BJ conducted the power analysis and performed statistical analysis. AB recruited the patients and performed surgery. MJ, DC, KHK, PC and MR analysed the data. MJ and DC wrote the manuscript. All other authors made critical revisions of the manuscript for intellectual content. All authors read and approved the final version of the manuscript.

## Appendix 1

### Details on statistical analysis

#### A) Power analysis

We prospectively performed a power analysis using the G*Power software, Version 3.1, to estimate the required sample size. As there were no preliminary data available on the specific question investigated here, we used the preoperative blood volume of a comparable collective published previously (*n *= 53; 4,123 ± 589 ml). For an estimated effect size we assumed an 'occult' decrease in blood volume during ANH that was considered to be relevant, but not yet detectable by hemodynamical changes (acute blood loss 'Class I', that is, 10 to 15% of the initial blood volume). Due to the high standardization of our setup, we assumed similar underlying physiological principles in comparable patients to lead to a high correlation among the repeated measurements. This led to the following parameters: predicted effect size d_z _= 1.06; α = 0.05; power level (1-β) = 0.90; correlation among repeated measurements r = 0.80. The required sample size was prospectively calculated to be ten.

After having investigated ten patients we repeated the calculation using the true values from our study. A maximum power level (1-beta) = 1.0 was shown due to the high and constant effect of ANH using ringer's lactate on blood volume (d_z _= 2.42) and the correlation among repeated measurements (r = 0.94).

#### B) Table 2

RCV: ANOVA: df = 2; F = 91.146; *P *= 0.000. Paired tests: measurement 1 vs. measurement 2: df = 9; T = 9.547; *P *= 0.000; measurement 1 vs. measurement 3: df = 9; T = 9.547; *P *= 0.000.

PV: ANOVA: df = 2; F = 17·596; *P *= 0·000. Paired tests: measurement 1 vs. measurement 2: n.s.; measurement 1 vs. measurement 3: df = 9; T = -3.150; *P *= 0.012; measurement 2 vs. measurement 3: df = 9; T = -7.286; *P *= 0.000.

BV: ANOVA: df = 2; F = 27.839; *P *= 0.000. Paired tests: measurement 1 vs. measurement 2: df = 9; T = 7.832; P = 0.000; measurement 1 vs. measurement 3: n.s.; measurement 2 vs. measurement 3: df = 9; T = -7.286; *P *= 0.000.

HK_WB_: ANOVA: df = 2; F = 46.221; *P *= 0.000. Paired tests: measurement 1 vs. measurement 2: df = 9; T = 5.861; *P *= 0.000; measurement 1 vs. measurement 3: df = 9; T = 7.317; *P *= 0.000; measurement 2 vs. measurement 3: df = 9; T = 6.708; *P *= 0.000.

HK_LV_: ANOVA: df = 2; F = 84.699; *P *= 0.000. Paired tests: measurement 1 vs. measurement 2: df = 9; T = 8.56.; *P *= 0.000; measurement 1 vs. measurement 3: df = 9; T = 9.795; *P *= 0.000; measurement 2 vs. measurement 3: df = 9; T = 7.606; *P *= 0.000.

HK_WB_/HK_LV _= F_cell_: ANOVA: df = 2; F = 26.191; *P *= 0.000. Paired tests: measurement 1 vs. measurement 2: df = 9; T = -6.159; *P *= 0.000; measurement 1 vs. measurement 3: df = 9; T = -5.841; *P *= 0.000; measurement 2 vs. measurement 3: n.s.

ESL: ANOVA: df = 2; F = 24.446; P = 0.000. Paired tests: measurement 1 vs. measurement 2: df = 9; T = 5.725; *P *= 0.000; measurement 1 vs. measurement 3: df = 9; T = 5.276; *P *= 0.001; measurement 2 vs. measurement 3: n.s.

Cumulative urine production: Paired tests: basal vs. measurement 2: df = 9; T = -10.065; *P *= 0.000; basal vs. measurement 3: df = 9; T = -16.389; *P *= 0.000; measurement 2 vs. measurement 3: df = 9; T = -5.268; *P *= 0.001.

Increase in interstitial water content: Paired tests: measurement 1 vs. measurement 2: df = 9; T = -11.261; *P *= 0.000; measurement 1 vs. measurement 3: df = 9; T = -7.996; *P *= 0.000; measurement 2 vs. measurement 3: df = 9; T = 4.629; *P *= 0.001.

#### C) Results section and Figure 2

Systolic arterial blood pressure: ANOVA: df = 2; F = 1.135; *P *> 0.05. Paired tests: measurement 1 vs. measurement 2: n.s.; measurement 1 vs. measurement 3: n.s.; measurement 2 vs. measurement 3: n.s.

Mean arterial blood pressure: ANOVA: df = 2; F = 2.567; *P *> 0.05. Paired tests: measurement 1 vs. measurement 2: n.s.; measurement 1 vs. measurement 3: n.s.; measurement 2 vs. measurement 3: n.s.

Diastolic arterial blood pressure: ANOVA: df = 2; F = 4.157; *P *= 0.027. Paired tests: measurement 1 vs. measurement 2: df = 9; T = 3.278; *P *= 0.01; measurement 1 vs. measurement 3: n.s.; measurement 2 vs. measurement 3: n.s.

Heart rate: ANOVA: df = 2; F = 1.12; *P *> 0.05. Paired tests: measurement 1 vs. measurement 2: n.s.; measurement 1 vs. measurement 3: n.s.; measurement 2 vs. measurement 3: n.s.

Catecholamine requirement: ANOVA: df = 2; F = 2.52; *P *> 0.05. Paired tests: measurement 1 vs. measurement 2: n.s.; measurement 1 vs. measurement 3: n.s.; measurement 2 vs. measurement 3: n.s.

As the number of patients was low (*n *= 10) the size effect was not checked for in particular, as verification of statistical significance by itself indicates it to be high under these conditions.

## Appendix 2

### Methodological details on double-label blood volume measurement

#### A) Determination of plasma volume

Immediately before each central venous dye injection, a two-point calibration was performed by measuring aliquots of the patient's blood containing two known indocyanine green (ICG) concentrations (ICG-Pulsion, Pulsion Medical Systems, Munich, Germany). The extinction of the blood was measured at both 800 and 900 nm in a spectrophotometer developed by our group. Subtracting extinction at 900 nm from that at 800 nm gives the specific ICG absorption. After calibration, 0.25 mg/kg of ICG was injected into the central venous catheter as a bolus dose over five seconds, the time of injection constituting time point 0. Between the second and the fifth minute after injection blood was continuously withdrawn from the arterial catheter through a cuvette attached to the spectrophotometer using a calibrated pump (approximately 20 ml/minute), and immediately reinfused. The ICG concentration at injection time was derived by monoexponential extrapolation of the curve of 19 extinction values between minute three and including minute five back to zero time. Measuring points were taken every 10 seconds. If values right at the beginning of the curve were not log-linear with respect to the following values, indicating possible incomplete mixing at these early times, the third minute was excluded from extrapolation. The criterion was the R^2 ^value of the initial six extinction values being under 0.99 in the semi-logarithmic plot. Positioning the extrapolated theoretical absorption value, at injection time, into the calibration curve yields CB_0 _- the theoretical whole-blood concentration of the dye at injection time. The corresponding theoretical plasma concentration (CP_0_) was then calculated as: CP_0 _= CB_0_/(1-HK_LV_), HK_LV _being the large vessel hematocrit. The values of PV were calculated as: PV (ml) = D (mg)/CP_0 _(mg × ml^-1^), D being the amount of dye injected.

#### B) Determination of red cell volume

For each measurement, 30 ml of whole blood was taken from a patient's peripheral vein to label red blood cells with sodium fluorescein (Fluorescein-Lösung 10%, Alcon Pharma, Freiburg, Germany). The blood was centrifuged and the red cell suspension incubated with 48 mg of sodium fluorescein for five minutes. To remove unbound fluorescein, the cells were washed twice using a calcium gluconate solution and then resuspended to the volume of the initial blood sample (30 ml) using Ringer's lactate.

The labelled red cells were reinfused via the central venous catheter. Three samples were drawn from the arterial catheter between the fifth and the tenth minute after injection, stored on ice, and analyzed in the laboratory by flow cytometry (FACScan, Becton Dickinson, Heidelberg, Germany) using an argon laser at 488 nm.

RCV was calculated according to: RCV (ml) = (E_i _× V_i _× HK_LV_)/(E_p _× F_Ef_), where E_i _= number of red cells injected per millilitre of labelled cell suspension, V_i _= volume of injected cell suspension (ml), HK = hematocrit of the subject's arterial blood (measured in triplicate), E_p _= number of red cells per millilitre in the patient's arterial blood (measured in triplicate), and F_Ef _= fraction of fluorescent erythrocytes as determined by flow cytometry.

The fraction of fluorescent red cells, determined by flow cytometry, was the mean value from determinations of all three drawn samples, each counted in triplicate as the number of fluorescent erythrocytes in 50,000 cells. E_i _and E_p _were obtained using a cell counter (530 nm; Coulter Electronics, Miami, FL, USA).

## Appendix 3

### Minor calculations from Table 1

Body surface area (BSA) was calculated according to a formula by Gehan and George [36]: BSA (m^2^) = 0.0235 × body weight (kg)^0.51456 ^× body height (cm)^0.42246^.

Body mass index (BMI) was calculated as: BMI (kg × m^-2^) = weight (kg) × (height (m))^-2^.
